# Extended interval BNT162b2 vaccination enhances peak antibody generation

**DOI:** 10.1038/s41541-022-00432-w

**Published:** 2022-01-27

**Authors:** Helen Parry, Rachel Bruton, Christine Stephens, Christopher Bentley, Kevin Brown, Gayatri Amirthalingam, Bassam Hallis, Ashley Otter, Jianmin Zuo, Paul Moss

**Affiliations:** 1 Institute of Immunology and Immunotherapy, University of Birmingham, Birmingham, B15 2TT UK; 2 National infection Service, Public Health England, Colindale, London, NW9 5EQ UK; 3 National infection Service, Public Health England, Porton Down, Salisbury, SP4 OJG UK

**Keywords:** Translational research, RNA vaccines

## Abstract

The BNT162b2 vaccine is highly effective against COVID-19 infection and was delivered with a 3-week time interval in registration studies^1^. However, many countries extended this interval to accelerate population coverage with a single vaccine. It is not known how immune responses are influenced by delaying the second dose. We provide the assessment of immune responses in the first 14 weeks after standard or extended-interval BNT162b2 vaccination and show that delaying the second dose strongly boosts the peak antibody response by 3.5-fold in older people. This enhanced antibody response may offer a longer period of clinical protection and delay the need for booster vaccination. In contrast, peak cellular-specific responses were the strongest in those vaccinated on a standard 3-week vaccine interval. As such, the timing of the second dose has a marked influence on the kinetics and magnitude of the adaptive immune response after mRNA vaccination in older people.

## Introduction

SARS-CoV-2 vaccines have demonstrated remarkable efficacy in protection against infection and symptomatic disease and offer the potential to provide large-scale protection against the COVID-19 pandemic^2^. The clinical severity of SARS-CoV-2 infection is increased in older people, and as such, this group has been prioritised for vaccination in most countries^2^. However, the quality of immune responses to vaccination deteriorates with age due to immunosenescence and understanding how to optimise vaccine schedules within this age group in order to maximise protection is a global priority^3^.

Real-world evidence now indicates that extending the interval between doses of BNT162b2 is highly effective^4^. Our previous work has shown that over 90% of people over the age of 80 years develop antibody responses at 5 weeks following a single BNT162b2 vaccine^5^. To understand the comparative immunological responses between the 2 different vaccine schedules following the second dose, we compared spike-specific antibody and cellular immune responses in 175 older people who underwent dual BNT162b2 vaccination, with either a 3-week or 11–12-week interval.

## Results

Participants aged 80 years and older, and who were living independently, were recruited to the study. The work was performed under the CIA UPH IRAS approval obtained from North West Preston ethics committee (REC 20/NW/0240) and conducted according to the Declaration of Helsinki and good clinical practice, with written informed consent obtained. In 99 participants the two doses were given at 3 weeks apart, which we term the “standard interval” (median age 84; IQR: 80–87, 42% male (42/99)). In 76 participants the two doses were given at 11–12 weeks apart which we term the “extended interval” (median age 84; IQR 82–89; 41% male (31/76)). No difference in the age of participants within the two cohorts was found (*p* = 0.320).

Previous natural infection with SARS-CoV-2 was confirmed through detection of nucleocapsid-specific antibodies and was present in 10 and 5 donors in the standard-and extended-interval vaccine regimens, respectively. As previous infection has a major impact on the immune response to vaccination, these donors were excluded from primary analysis.

Venepuncture blood samples were taken at two timepoints. Within the standard-interval cohort, the first sample was taken at 2–3 weeks following the second dose to determine the “peak response” to the vaccine boost. A second sample was taken 8–9 weeks after the second dose in order to assess the stability of antibody and cellular responses over this period (*n* = 79). Informed consent was formally obtained from every donor. These vaccination schedules were standard of care and this work was not undertaken as a formal clinical trial.

**Fig. 1:**
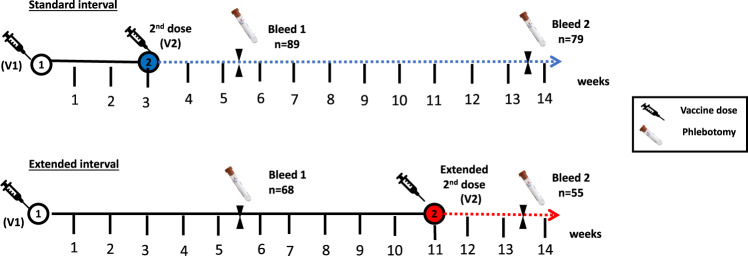
Infographic to show vaccination timings and blood collection. Blood samples were obtained on all participants at 5–6 weeks (2–3 weeks post second dose of vaccine (V2) for the standard interval cohort and 5–6 weeks post first dose of vaccine (V1) in the extended interval cohort). Blood samples were obtained again at 13–14 weeks post first dose of vaccine in both cohorts (coinciding, 10–11 weeks post second vaccine (V2) in the standard-interval cohort and 2–3 weeks post the second dose of vaccine (V2) in the extended-interval cohort). Numbers of study participants shown represent the analysis cohort following exclusion of participants with previous natural infection.

For donors with an extended-interval vaccine schedule, blood samples were taken at 5–6 weeks following the first dose (*n* = 68) and then again at 8–9 weeks later. This second time point was 2–3 weeks after the second vaccine dose and therefore represented the “peak response” in the extended-interval cohort (*n* = 55) (Fig. 1). These time points were contemporaneous with the standard-interval cohort allowing a direct comparison of the immunological outcomes over a fixed period of time.

Spike-specific antibodies were detected in 100% of participants in the standard-interval cohort at both the first and second timepoints (*n* = 86 and *n* = 75, respectively). Within the extended-interval cohort, antibodies were detectable in 91% (62/68) at the first timepoint, at 5–6 weeks after the first dose, but this rose to 100% 2–3 weeks after the second dose.

**Fig. 2:**
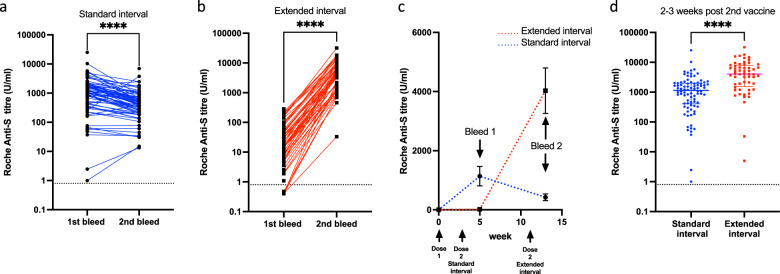
An extended interval to the second vaccination of BNT162b2 stimulates stronger spike-specific antibody responses. **a** Dot plot to compare spike-specific antibody responses by Roche platform in participants who obtained the BNT162b2 vaccine with a standard interval of 3 weeks apart at bleed timepoint 1 (2–3 weeks post vaccine dose 2) and timepoint 2 (10–11 weeks post vaccine dose 2) (Wilcoxon matched-pair signed-rank test, *p* < 0.0001). **b** Dot plot to compare spike-specific antibody responses by Roche platform in participants who obtained the BNT162b2 vaccine with an extended interval at bleed timepoint 1 (5–6 weeks post vaccine dose 1) and bleed timepoint 2 (2–3 weeks post vaccine dose 2) (Wilcoxon matched-pair signed-rank test, *p* < 0.0001). **c** The kinetics of the anti-spike antibody of the 2 different vaccine schedules are shown over a 14-week period. Those participants who obtained the BNT162b2 vaccine with a standard 3-week interval are shown in blue, while those who received an extended interval to second-dose vaccination of BNT162b2 are shown in red (SEM is shown). **d** Dot plot to compare spike-specific antibody responses by Roche platform in the participants 2–3 weeks after the second dose of BNT162b2 vaccination in the standard- and extended-interval cohorts (median and IQR shown) (Mann–Whitney U test, *p* < 0.0001).

We next went on to assess the magnitude of the antibody response at the two timepoints within both cohorts. Antibody titres in the standard-interval regimen peaked at 1138 U/ml after the second dose and then fell by 2.6-fold over the subsequent weeks (*p* < 0.0001) (Fig. 2a). Within the extended-interval cohort, the median antibody titre was 17 U/ml at 5–6 weeks weeks after the first dose, but showed a substantial 242-fold increase to reach 4030 after the second dose (*p* < 0.0001) (Fig. 2b). The kinetics of antibody responses in the standard- and extended-interval cohorts are shown graphically in Fig. 2c.

When peak antibody responses after the second dose were contrasted in both cohorts, it was apparent that values in the extended-interval group were 3.5-fold higher at 4030 U/ml compared with 1138 U/ml (*p* = <0.0001; Fig. 2d).

Samples were next analysed on the MSD platform that assesses antibody responses against the whole spike protein and the receptor-binding domain (RBD). RBD-specific binding is valuable as the majority of neutralising antibodies bind within this region. Here we also found a higher spike-specific response following the second dose within donors in the “extended interval” cohort (median 69,520 U/ml vs 59,910 in “standard cohort”; *p* = 0.005) (Supplementary Fig. 1). Antibody titres against RBD at this timepoint were also higher in the extended-interval cohort (median 41,460 U/ml vs 37,840 U/ml after two doses in the standard-interval cohort, *p* = 0.017).

We were also interested to understand if cellular immunity differed between the two regimens and for this we utilised an interferon-gamma (IFN-γ) ELISPOT assay, in order to determine spike-specific T-cell responses following vaccination in the two cohorts. Cellular responses against two peptide pools from the S1 and S2 spike domains were determined following overnight stimulation. Values from both wells were aggregated to give the total spike-specific response per million peripheral blood mononuclear cells.

**Fig. 3:**
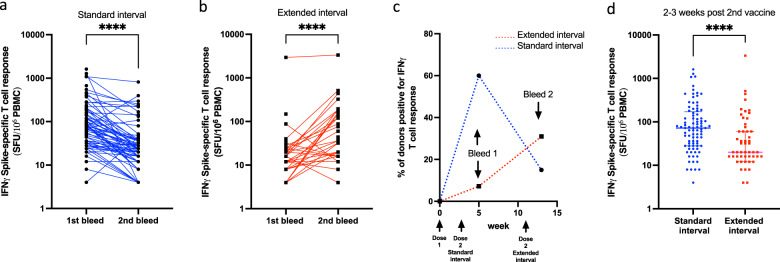
Standard-interval vaccination with BNT162b stimulates a greater peak cellular response. **a** Dot plot to compare spike-specific cellular responses by IFN-γ ELISpot in participants who obtained the BNT162b2 vaccine with a standard interval of 3 weeks apart at bleed time point 1 (2–3 weeks post vaccine dose 2) and timepoint 2 (10–11 weeks post vaccine dose 2) (Wilcoxon matched-pairs signed rank test; *p* < 0.0001). **b** Dot plot to compare spike-specific antibody responses by IFN-γ ELISpot in participants who obtained the BNT162b2 vaccine with an extended interval at bleed timepoint 1 (5–6 weeks post vaccine dose 1) and timepoint 2 (2–3 weeks post vaccine dose 2) (Wilcoxon matched-pair signed-rank test). **c** The percentage of donors with a positive IFN-γ ELISpot T-cell response is shown in the two different vaccine-interval cohorts over a 14-week period. The percentage response of those donors receiving the vaccine doses on a standard interval are shown in blue, while those receiving it on an extended interval are shown in red. **d** Dot plot to compare spike-specific cellular responses by ELISpot in the participants 2–3 weeks after the second dose of BNT162b2 vaccination in the standard- and extended-interval cohorts (median and IQR shown) (Mann–Whitney U test, *p* < 0.0001).

Within the standard-interval cohort 60% (53/88) of donors had a confirmed cellular response at 2–3 weeks following the second dose although this fell to only 15% (12/78) 8–9 weeks later. The proportion of participants demonstrating a cellular response in the extended-interval cohort was only 7% (5/68) at 5–6 weeks after the first dose, but this rose to 31% (17/55) 2–3 weeks after the second dose (Fig. 3c).

The magnitude of spike-specific T-cell responses in the standard-interval cohort peaked at 72 spots/million PBMC at 2–3 weeks following the second dose and then fell by 3-fold to 24 spots/million after 8–9 weeks (*p* < 0.0001) (Fig. 3a). Within the extended-interval cohort these corresponding values were 8 spots/million 5–6 weeks after the first dose, followed by a 2.5-fold rise to reach 20 spots/million 2–3 weeks after the second dose (*p* < 0.0001) (Fig. 3b).

A comparison of the median magnitude of peak cellular responses after the second dose in the two schedules showed that these were higher for donors in the standard-interval regime (72 vs 20 spots/million, *p* < 0.0001) (Fig. 3d).

Cellular immune responses play an important role in supporting and maintaining antibody production and we therefore assessed the relationship between the cellular response 2 weeks following the second dose and the degree of subsequent antibody waning over the next 8–9 weeks in the standard-interval cohort. No association was found between S1- and S2-specific cellular responses and the rate of decline in antibody titre (*r* = −0.01, *p* = 0.93) (Supplementary Fig. 2).

## Discussion

Extended-interval vaccination has been adopted in several countries and shown good clinical efficacy^4^. Here we show that this approach leads to an enhanced peak antibody response after the second BNT162b2 vaccine dose although peak cellular responses were lower. These observations raise a number of questions regarding the underlying mechanisms of adaptive immunity to vaccination with potential relevance to vaccine strategy.

Spike-specific antibody responses within the first 2–4 weeks after the second dose of COVID-19 vaccine are emerging as a potential immune correlate of protection after vaccination^6^. As such, the strong humoral responses that are elicited by mRNA vaccines are likely to underlie their excellent clinical efficacy to date^7^. Our findings confirm previous studies showing that the 3-week standard-interval BNT162b2 regimen elicits strong antibody responses in older people^8^. As previously reported, we detected antibody responses in all donors at 2 weeks after the second vaccine. Here we were able to extend this work to assess the stability of adaptive immunity over the next two months. A median 2.6-fold reduction in antibody titre was seen over this 8-week period and indicates waning in the early post-boost period. However, systemic antibody levels would be expected to subside within the first few weeks following antigen challenge and absolute values remained substantial in most people^9^. It will be important to assess how antibody levels are maintained over longer periods and this is likely to define the potential need for booster vaccines in this vulnerable age group.

There is no information to date regarding the impact of extended-interval mRNA vaccination on peak antibody responses after the second dose in older people. Interestingly, we found that this approach boosted median peak antibody titres by 3.5-fold compared with those seen after standard regimen. Very high antibody levels were seen in a substantial proportion of donors with remarkable titres up to 18,100 U/ml. These responses are encouraging for long term protection and, although expected to reduce substantially over the subsequent few weeks, it is possible that this higher baseline may act to provide more robust long-term protection. This may be of particular importance in relation to protection from viral variants of concern which may emerge as a major challenge for COVID-19 vaccinees^10^. It will be of interest to assess the relative induction of long-lived plasma cells and memory B cells following each regimen in order to assess potential cellular correlates of antibody response^11^.

Prior natural infection with SARS-CoV-2 strongly enhances vaccine responses and median peak antibody responses of 32,250 U/ml were seen in the 10 donors who received the standard-interval vaccination protocol. Of note, these values fell by 3.5-fold over the subsequent 8 weeks (32250 after the 1^st^ bleed vs 9235 at the 2^nd^ bleed U/ml, *p* = 0.002), which is a greater rate of decline than noted in the infection-naive cohort where a 2.6-fold decrease was found. This requires further longitudinal follow-up in order to see if antibody levels plateau at values higher than in infection-naive donors. Of the 5 donors in the extended-interval protocol who had prior natural infection, 2 had blood sampling at the second bleed point, with peak antibody responses of 90,750 U/ml.

The importance of cellular immunity in providing clinical protection against SARS-CoV-2 is not currently clear. Our findings suggest that earlier administration of the second dose of vaccine provides a greater boost to the cellular immune response. It is not clear why antibody and cellular responses show a differential response to standard and extended-interval vaccination. mRNA vaccines lead to germinal centre formation and particularly strong induction of antibody responses^11,12^, but somewhat less is known regarding the induction of cellular immunity^13^.

In both cohorts, the proportion and magnitude of T-cell response were somewhat lower than those reported in younger cohorts and may reflect the impact of immunosenescence^14^. It should be noted that T-cell responses were measured on the standard assessment of IFN-γ secretion, but this does not preclude the presence of spike-specific T cells, which make other inflammatory cytokines and may be induced preferentially following mRNA vaccination^15^. In particular, induction of T-follicular helper cells often correlates with antibody induction. Indeed, one role for cellular immunity following SARS-CoV-2 vaccination may be to support the generation and maintenance of antibody, but we did not find any evidence to suggest that the magnitude of cellular response following the second vaccine was associated with the rate of waning of antibody responses in the standard-interval cohort.

Longitudinal analysis is planned on this cohort and will allow assessment of the relative importance of memory B-cell and T-cell populations and functional neutralisation activity. These data will enhance understanding of the impact of dosing interval on longer-term immune function.

Recent studies indicate that antibody levels remain robust for 6 months following the 3-week double-mRNA vaccination and decline with a half-life of 52 days after day 43^16^. This is likely to underlie the impressive extended clinical efficacy over this time period^17^. Our findings raise the question of whether or not the clinical efficacy of dual-mRNA vaccination might be further enhanced by extending the interval between doses. It is noteworthy that an extended-interval protocol for the adenovirus-based ChAdOx1 vaccine has also been shown to increase spike-specific antibody responses by 2.3-fold and to improve vaccine efficacy^18^. Enhanced antibody generation has also been reported in younger healthcare workers with the BNT162b2 vaccine^19^ The potential disadvantage of this approach is that it extends the period of partial protection prior to the second dose. However, epidemiological data indicate that single vaccination delivers strong clinical protection against symptomatic COVID-19 infection, and this may therefore not represent a major concern^4,20^. As such, if extended vaccine schedules act to establish a higher “baseline” level of SARS-CoV-2-specific antibody, then this may be worthy of consideration in relation to potentially minimising the need for subsequent revaccination.

In conclusion, we show that extended-interval vaccination with BNT162b2 increases the peak antibody response by 3.5-fold in older people. This may help to sustain humoral immunity over the longer term and further improve the clinical efficacy of this powerful vaccine platform.

## Methods

### Participants

All 175 participants were 80 years or older and received 2 doses of the BNT162b2 Pfizer/BioNTech vaccine. Participants were vaccinated with either a standard 3-week interval between doses or an extended-interval schedule, with the second vaccine dose given 11–12 weeks after the first dose. These schedules were clinical standard of care. Participants were recruited at the vaccination centre and formal consent was obtained in every case. No further clinical or demographic data were obtained. Participants received the same phlebotomy time points at 5–6 weeks and 13–14 weeks following the first vaccine, for comparative purposes. Samples were taken by a trained phlebotomist in the home setting.

### Roche Elecsys^®^ electrochemiluminescence immunoassay (ECLIA)

Serum was stored at −20 °C and defrosted prior to antibody analysis. IgG/A/M antibodies specific to SARS-CoV-2 were detected using electrochemiluminescence assays on the automated Roche Cobas e801 analysers based at Public Health England (PHE) Porton. Calibration and quality control were performed as recommended by the manufacturer. Antinucleocapsid protein (NP) antibodies were detected using the qualitative Roche Elecsys^®^ AntiSARS-CoV-2 ECLIA (COV2, Product code: 09203079190), while antispike (S) antibodies were detected using the quantitative Roche Elecsys^®^ Anti-SARS-CoV-2 S ECLIA (COV2 S, Product code 09289275190). Antinucleocapsid results are expressed as cutoff index (COI) value, with a COI value of ≥ 1.0 considered positive for antinucleocapsid antibodies. Antispike results are expressed as units per ml (U/ml), with samples with a result of ≥0.8 U/ml considered positive for anti-spike antibodies within the fully quantitative range of the assay: 0.4–2500 U/ml. Samples >2500 U/ml were diluted further (1:10, 1:100, and 1:1000) to within the quantitative range.

### Mesoscale-discovery (MSD) IgG assay

Quantitative IgG antibody titres were measured against spike (S) protein, nucleocapsid protein (N), and other antigens using the MSD V-PLEX COVID-19 Respiratory Panel 2 (96-well, 10 Spot Plate was coated with three SARS CoV-2 antigens (S, S-RBD S-NTD, and N)) (Cat # K15372U, Lot # Z0056764) from Meso Scale Diagnostics, Rockville, MD USA (Appendix) and ran in duplicate. Antigens were spotted at 200–400 μg/mL. Multiplex MSD assays were performed as per the instructions of the manufacturer. To measure IgG antibodies, 96-well plates were blocked with MSD Blocker A for 30 min. Following washing with buffer, the samples were diluted 1:500 in diluent. Reference standards and positive controls and diluted samples were added to the wells. After 2-hour incubation and plates were washed 3x with wash buffer and detection antibody (MSD SULFO-TAG™ Anti-Human IgG Antibody, 1/200) diluted in diluent 100 was added. After 1 h of incubation at RT, the plates were washed 3x with wash buffer. MSD GOLD™ Read Buffer B was added and plates were read immediately using a MESO TM QuickPlex SQ 120. Text files were then generated from the Methodical Mind software and transferred to the MSD Discovery Workbench (v4.0) software. Data were then converted to AU/ml and exported as .csv files. The values from exported data were adjusted for any sample dilution.

### Cellular assays

Peripheral blood mononuclear cells (PBMCs) were isolated from a whole-blood sample using “T-Cell Xtend” (Oxford Immunotec) and Ficoll. After quantification and dilution of recovered cells, 250,000 PBMCs were plated into each well of a “T-SPOT Discovery SARS-CoV-2” kit (Oxford Immunotec). This is designed to measure responses to overlapping peptide pools covering protein sequences of four different SARS-CoV-2 antigens, without HLA restriction, and includes negative and positive controls. Peptide sequences that showed high homology to endemic coronaviruses were removed from the sequences, but sequences that may have homology to SARS-CoV-1 were retained. Cells were incubated and interferon-γ-secreting T cells were counted. A cutoff of 6+ spots per 250,000 PBMCs on the S1 pool was defined as a positive response in line with the Oxford Immunotec diagnostic Covid kit and are presented in the text.

### Statistical analysis

The data set was first tested for normality using Kolmogorov–Smirnov analysis. Nonparametric analysis was used throughout. For comparative analysis of antibody titres and cellular responses within the same cohort, Wilcoxon ranked pairs was performed. For comparative analysis of antibody or cellular responses between the 2 cohorts, two-sided Mann–Whitney U-test was performed. Two-sided Spearman’s rank correlation was used to assess the relationship with cellular response and the rate of antibody waning. All analysis was performed using Graphpad Prism v9.1.0 for Mac (San Diego, California, USA).

### Reporting summary

Further information on research design is available in the Nature Research Reporting Summary linked to this article.

## Supplementary information

Suuplementary

REPORTING SUMMARY

## Data Availability

The datasets generated/analysed during the current study are available from the corresponding author upon reasonable request.

## Code availability

No coding was used for this work.
