# Supplementary material for: Extended interval BNT162b2 vaccination enhances peak antibody generation
Source: NPJ Vaccines. 2022 Jan 27;7:14. doi: 10.1038/s41541-022-00432-w (PMC8795435; doi:10.1038/s41541-022-00432-w)
Supplement: Supplementary file 1 — Suuplementary [file 41541_2022_432_MOESM1_ESM.pdf]

Supplementary Figure 1

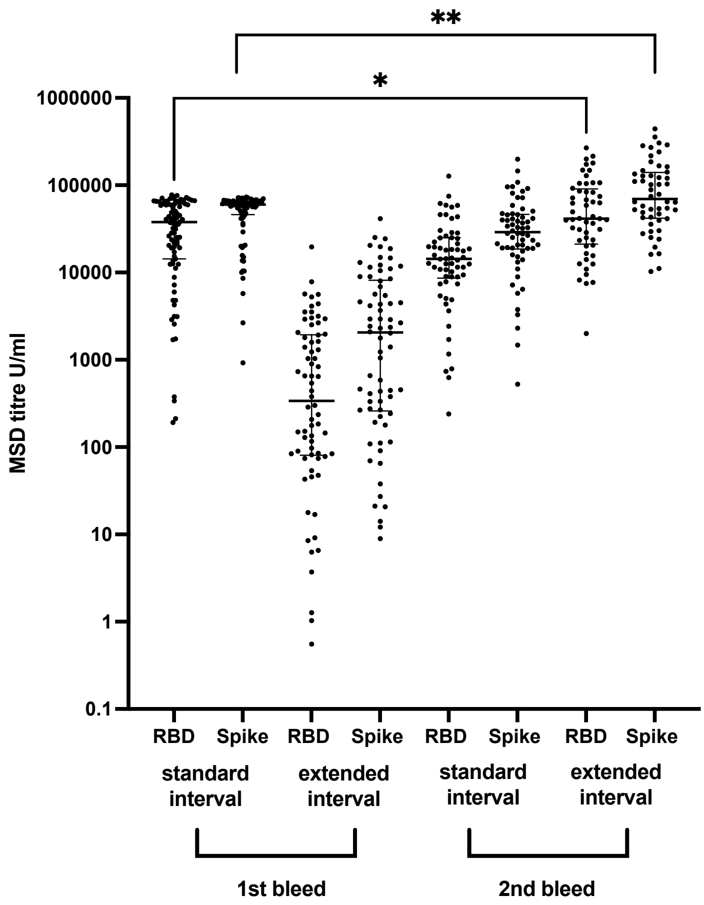

Spike-specific and Receptor Binding Domain (RBD)-specific antibody responses amongst participants on the standard and extended interval cohorts at the two sampling timepoints are shown. Increased responses were observed in the extended interval for total Spike (Mann-Whitney p=0.005) and RBD (Mann-Whitney p=0.017) compared to the standard interval cohort.

Supplementary Figure 2

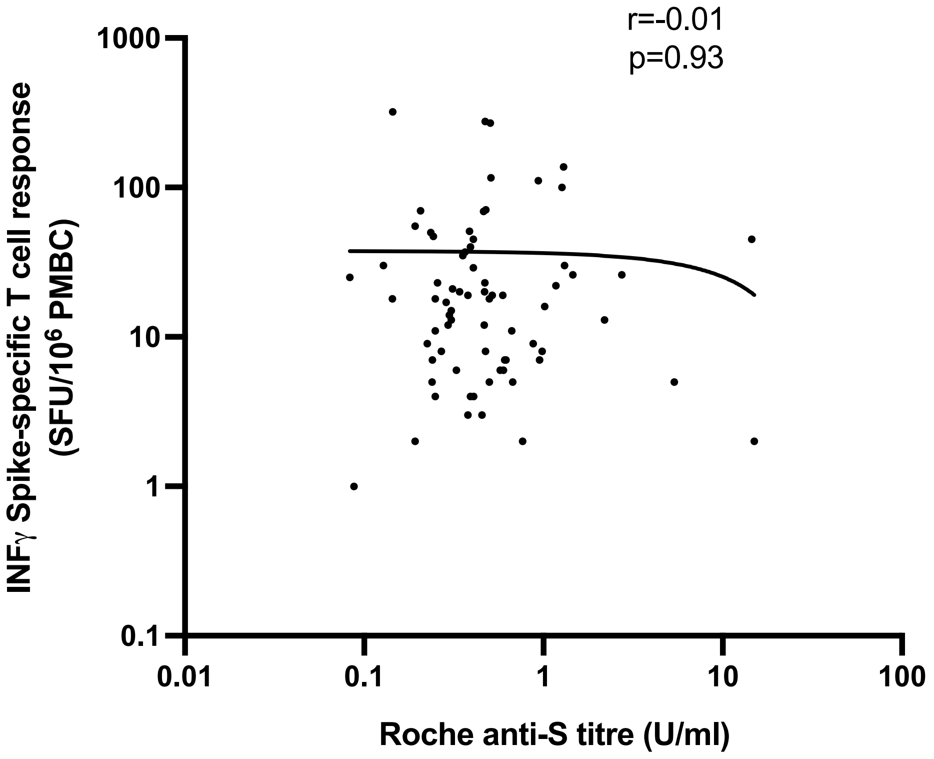

Correlation between spike-specific T cell responses and spike-specific antibody titres amongst donors on the standard interval cohort at the second sampling time point is shown (Spearman's rank correlation co-efficient r=-0.01; p=0.93).
