# Supplementary material for: Extended interval BNT162b2 vaccination enhances peak antibody generation
Source: NPJ Vaccines. 2022 Jan 27;7:14. doi: 10.1038/s41541-022-00432-w (PMC8795435; doi:10.1038/s41541-022-00432-w)
Supplement: Supplementary file 2 — REPORTING SUMMARY [file 41541_2022_432_MOESM2_ESM.pdf]

nature research

Corresponding author(s): Paul Moss

Last updated by author(s): Oct 11, 2021

Reporting Summary

Nature Research wishes to improve the reproducibility of the work that we publish. This form provides structure for consistency and transparency in reporting. For further information on Nature Research policies, see our Editorial Policies and the Editorial Policy Checklist.

Statistics

For all statistical analyses, confirm that the following items are present in the figure legend, table legend, main text, or Methods section.

n/a	Confirmed	
☐	☒	The exact sample size (n) for each experimental group/condition, given as a discrete number and unit of measurement
☒	☐	A statement on whether measurements were taken from distinct samples or whether the same sample was measured repeatedly
☐	☒	The statistical test(s) used AND whether they are one- or two-sided<br>*Only common tests should be described solely by name; describe more complex techniques in the Methods section.*
☒	☐	A description of all covariates tested
☐	☒	A description of any assumptions or corrections, such as tests of normality and adjustment for multiple comparisons
☐	☒	A full description of the statistical parameters including central tendency (e.g. means) or other basic estimates (e.g. regression coefficient) AND variation (e.g. standard deviation) or associated estimates of uncertainty (e.g. confidence intervals)
☐	☒	For null hypothesis testing, the test statistic (e.g. F, t, r) with confidence intervals, effect sizes, degrees of freedom and P value noted<br>*Give P values as exact values whenever suitable.*
☒	☐	For Bayesian analysis, information on the choice of priors and Markov chain Monte Carlo settings
☒	☐	For hierarchical and complex designs, identification of the appropriate level for tests and full reporting of outcomes
☐	☒	Estimates of effect sizes (e.g. Cohen's d, Pearson's r), indicating how they were calculated

Our web collection on statistics for biologists contains articles on many of the points above.

Software and code

Policy information about availability of computer code

Data collection: ELISPOT data was collected by Oxford Immunotec Ltd and Roche antibody data was collected by Public Health England, Porton Down. MSD data was collected at University of Birmingham.

Data analysis: Statistic analysis was carried out with GraphPad Prism v9.1.0 for Mac (San Diego, California USA). MSD discovery workbench (v4.0) was used for MSD data analysis.

For manuscripts utilizing custom algorithms or software that are central to the research but not yet described in published literature, software must be made available to editors and reviewers. We strongly encourage code deposition in a community repository (e.g. GitHub). See the Nature Research guidelines for submitting code & software for further information.

Data

Policy information about availability of data

All manuscripts must include a data availability statement. This statement should provide the following information, where applicable:

- Accession codes, unique identifiers, or web links for publicly available datasets
- A list of figures that have associated raw data
- A description of any restrictions on data availability

The datasets generated during the current study are available from the corresponding author upon reasonable request.

Field-specific reporting

Please select the one below that is the best fit for your research. If you are not sure, read the appropriate sections before making your selection.

☒ Life sciences ☐ Behavioural & social sciences ☐ Ecological, evolutionary & environmental sciences

For a reference copy of the document with all sections, see nature.com/documents/nr-reporting-summary-flat.pdf

Life sciences study design

All studies must disclose on these points even when the disclosure is negative.

Sample size	The sample size was not determined using statistical calculation. This is unknown territory and we recruited as many donors as possible in the vaccination centres whilst operating during a national lockdown.
Data exclusions	Only samples from oxford immuotec Ltd with insufficient IFN gamma production in the positive control (PHA) were excluded.
Replication	The ELISPOT was carried out in triplicate. MSD was carried out in duplicate.
Randomization	The two groups compared in the study were determined according to their vaccine interval duration which was assigned outside of the study.
Blinding	Investigators were blinded on which samples were from which vaccine interval group.

Reporting for specific materials, systems and methods

We require information from authors about some types of materials, experimental systems and methods used in many studies. Here, indicate whether each material, system or method listed is relevant to your study. If you are not sure if a list item applies to your research, read the appropriate section before selecting a response.

Materials & experimental systems

n/a	Involved in the study	
☒	☐	Antibodies
☒	☐	Eukaryotic cell lines
☒	☐	Palaeontology and archaeology
☒	☐	Animals and other organisms
☐	☒	Human research participants
☒	☐	Clinical data
☒	☐	Dual use research of concern

Methods

n/a	Involved in the study	
☒	☐	ChIP-seq
☒	☐	Flow cytometry
☒	☐	MRI-based neuroimaging

Human research participants

Policy information about studies involving human research participants

Population characteristics	The donors' average age is 84 years in both cohorts with 42% and 41% respectively being male sex for standard and extended interval groups. No further demographic data is available.
Recruitment	Recruitment took place in local vaccination centres and through invitation via local primary networks.
Ethics oversight	This study was approved under CIA UPH IRAS obtained from North West Preston ethics committee (REC 20/NW/0240)

Note that full information on the approval of the study protocol must also be provided in the manuscript.
